# Long-term frailty trajectory in older ICU survivors requiring mechanical ventilation: a prospective cohort study in Norway

**DOI:** 10.1016/j.aicoj.2026.100115

**Published:** 2026-07-16

**Authors:** B.A. Kroken, D. Bergum, K.S. Berg, M. Espinasse, O.K. Fossum, M. Hoff, P. Klepstad, B.Å. Sjøbø, R. Kvåle, H. Flaatten, S.K. Frisvold

**Affiliations:** a Department of Clinical Medicine, UiT the Arctic University of Norway, Tromso, Norway; b Department of Anaesthesia and Intensive Care, University Hospital of North Norway, Tromso, Norway; c Department of Anaesthesiology and Intensive Care Medicine, St Olavs University Hospital; d Department of Anaesthesia and Intensive Care Medicine, Surgical Clinic, Nordland Hospital Trust, Bodø, Norway; e Faculty of Nursing and Health Sciences, Nord University, Bodø, Norway; f Department of Clinical Research, University Hospital of North Norway, Tromso, Norway; g Department of Anaesthesia and Intensive Care, Akershus University Hospital, Oslo, Norway; h Department of Surgical Services, Intensive Care Unit, Haukeland University Hospital, Bergen, Norway; i Department of Clinical Medicine, University of Bergen, Norway; j Department of Anaesthesia and Intensive Care, Haukeland University Hospital, Bergen, Norway; k Department of Research and Development, Haukeland University Hospital, Bergen, Norway

**Keywords:** Frailty, Intensive care, Mortality, Critical illness, Elderly

## Abstract

**Background:**

While intensive care unit (ICU) capacity remains constrained, the number of older patients receiving resource-demanding ICU treatment continues to rise. Yet, long-term outcomes and post-critical illness frailty trajectories remain poorly characterized. We examined long-term mortality and frailty trajectories, and their associations with clinical characteristics and ICU treatment intensity.

**Methods:**

In this prospective, observational study across five Norwegian hospitals representing all health regions, we included patients aged ≥65 years who required invasive mechanical ventilation for ≥24 h. Frailty was assessed using the Clinical Frailty Scale (CFS) at baseline and at 3 and 12 months, categorized as robust (CFS 1–3), prefrail (CFS 4) and frail (CFS ≥ 5). Multiple regression models were used to examine associations between frailty trajectories and mortality, with baseline frailty, clinical characteristics and potentially modifiable aspects of ICU treatment.

**Results:**

346 patients were included. Mean age was 74 years (± 6), and 98 % lived at home before admission. The median ICU length of stay was 8 (IQR 9) days. Total mortality was 52 %, with most deaths occurring before hospital discharge. In the survivors, the prevalence of frailty increased from 23 % (79/346) at baseline, to 47 % (81/174) at three months. Between the three- and 12- month follow-up, CFS scores remained unchanged in 71 % (108/153), improved in 19 % (29/153) and deteriorated in 10 % (15/153). By 12 months 86 % (132/153) had returned home, though 32 % (49/153) required home care compared to 10 % pre-admission. Baseline CFS showed the strongest association with 12-month mortality (Risk Ratio 1.46, *p <* 0.001). Age (Risk Ratio 1.22, *p <* 0.001) and SOFA score on the first day after intubation (Risk Ratio 1.14, *p <* 0.01) were also significant predictors, whereas comorbidity was not.

**Conclusions:**

In elderly ICU-patients long-term frailty is deranged, and baseline CFS is closely associated with mortality. However, a small group of patients had a transitory decline in frailty at three months follow-up and then improved. Intervention studies targeting modifiable factors associated with long-term functional decline and mortality are warranted.

**Trial registration:**

ClinicalTrials.gov (NCT06012942).

## Highlights

• Nearly all older ICU patients came from home, yet half were pre-frail/frail.
• Nine out of ten deaths occur within the first 3 months of critical illness.
• Post-ICU frailty doubled within 3 months, followed by subacute recovery.
• Nearly all survivors returned home at 12 months, though home care tripled.
• Pre-illness frailty predicts long-term mortality better than age or comorbidity.

## Background

As more elderly patients require intensive care, a growing number of survivors face significant long-term impairments. Specifically, this population frequently suffers from physical impairments including sarcopenia and loss of muscle integrity, cognitive deficits ranging from memory loss to dementia and psychological impairments including anxiety and depression; recognised as post-intensive care syndrome [1]. Apart from severely diminishing patients´ quality of life, the burden of this syndrome even extends to their families with 30–70 % experiencing long-term distress [1,2]. Furthermore, the adverse morbidity outcomes lead to increased healthcare utilization post-ICU, including readmissions and institutionalisation [3]. These challenges underscore the need to develop generally applicable criteria for triage decisions and treatment strategies in the elderly [4,5]. Currently, triage and treatment selection often rely on individual clinical judgement, and therefore vary mainly according to age and intensive care unit (ICU) capacity [5,6].

Recent studies have shown that frailty, more than age and comorbidity, might be the most important determinant of survival, physical and cognitive outcomes in the ICU population [[7], [8], [9]]. The frailty approach provides a comprehensive assessment of patient vulnerability, by containing functional capacity, daily activity and social support [10]. The Clinical Frailty Scale (CFS) is at present considered the most appropriate tool for assessing frailty in the ICU setting [[11], [12], [13], [14]].

Although frailty predicts ICU survival, long-term outcomes in elderly patients, with respect to the progression of frailty and associated risk factors, are still less defined [1]. This study primarily aims to determine changes in CFS from pre-admission to three and 12 months after ICU in patients ≥65 years of age who require mechanical ventilation. Secondary aims include evaluating how demographics, comorbidities, disease severity, treatment intensity, and baseline CFS influence frailty trajectories and mortality.

## Methods

### Study design and participants

This is a prospective longitudinal observational study including patients from five general ICUs located in all health care regions of Norway: Akershus University hospital, Haukeland University hospital, St Olav University hospital, Nordland hospital and the University hospital of North Norway. The trial protocol has been published previously [15].

All admitted patients were screened by local study participants. Inclusion began between July 2022 and January 2023 and was terminated in the last centre July 2024. Inclusion criteria were age ≥ 65 years and need for invasive mechanical ventilation for ≥24 h. Exclusion criteria were lack of written consent from the patient or next of kin, residency outside of Norway, and inability to complete the questionnaire due to cognitive/physical limitations and the absence of next of kin.

The study was performed according to the Strengthening the reporting of observational studies in epidemiology (STROBE) checklist [16].

### Frailty

The Norwegian translation of the CFS version 2.0 pictogram was used to assess frailty in the study [11,17]. The CFS consists of 9 levels. In our analysis, frailty level was divided into three groups. CFS 1–3 (robust) are individuals having no limitations of illness. CFS 4 (prefrail) represents a transitional state where individuals exhibit lower physiological reserve due to aging or illness, resulting in a lower stress tolerance. CFS 5–9 (frail) are individuals with established functional limitations who face a greater risk of multi-domain deterioration when exposed to stressors such as critical illness [8,18].

### Data collection

Demographic and clinical data were retrieved from the medical records. Comorbidity was assessed with Comorbidity Polypharmacy Score (CPS). The CPS is rated in clusters where 0–7 is mild, 8–14 is moderate, 15–21 is severe, and >21 is morbid comorbidity [7]. Residence and discharge destination was categorized to three groups: no home nursing care, home nursing care, and institution.

Frailty was assessed by dedicated researchers who had completed standardised training in the use of the CFS and followed published guidelines [11]. Assessment was conducted in collaboration with the patient or the next of kin. The baseline CFS intended to describe the habitual status before critical illness, often set to two weeks prior to ICU admission.

Data on mortality was retrieved from the Norwegian Population Registry [19]. Time of death was clustered in ICU-, hospital-, 30 days, three months- and 12 months-mortality, defined by days after inclusion. The cause of death was obtained from medical journal or local health providers.

Three and 12 months following inclusion, the patient or their next of kin were contacted by mail or email and provided with study information and the CFS pictogram. This was followed by a structured telephone interview conducted by one of four study team members. During the interview, CFS, comorbidities, residence, rehabilitation stay, hospital and ICU readmissions were recorded. Follow-up was conducted within a predefined window, from one week before to two weeks after the three- and 12-month time points.

Due to a late protocol amendment, two additional survey items were administered to only the final 68 ICU survivors; this cohort was consequently designated as an exploratory pilot sub-analysis. At 12-month follow-up, they were asked whether they considered their quality of life as unchanged, improved or declined, compared to their pre-critical illness state, and whether they would consent to similar ICU-admission in the future if necessary.

Study data were aggregated and stored de-identified in REDCap eCRF (Research Electronic Data Capture) on a secure data server [20].

### Outcome

The primary outcome was the CFS score trajectory, defined as the difference between the pre-critical illness (baseline) CFS and CFS measured at three and 12 months after study inclusion.

Secondary outcomes were the CFS at three and 12 months, mortality during the study period, associations between baseline CFS and mortality, and associations between demographic, clinical, and ICU-related variables with CFS trajectory and mortality.

### Statistical analysis

The sample size calculation was based on detecting a between-group difference of 0.25 in the CFS, assuming a power of 80 % and a two-sided alpha level of 0.05. The effect size estimate was derived from data reported in a previous Irish study [21]. On this basis, a total sample size of 350 participants was required.

Normally distributed continuous data were described as means with standard deviation. Non-normally distributed continuous data were described as median with interquartile range. Categorical data were presented as counts and percentage.

Normality in the continuous data was visually assessed in plots and by using the Shapiro–Wilk test.

Differences between groups and between baseline and follow-up were analysed using the Pearson Chi square test for categorical variables, Student’s t-test and one-way ANOVA for continuous variables. Assumptions of normality and homogeneity of variance were assessed prior to parametric testing. The following candidate variables were examined using univariable analyses and visual analysis to explore association with mortality and change in CFS: age, gender, body mass index, CPS, baseline residence, discharge destination, need for stay in institution at three and 12 months, hospital length of stay (LOS), ICU LOS, ventilator-time, need for renal replacement therapy (RRT), rehabilitation-stay, readmissions to ICU and hospital, and baseline CFS.

Multivariable linear regression was used to evaluate predictors of change in CFS, and multivariable logistic regression was used to identify predictors of 12-month mortality.

For linear regression models, assumptions of linearity, normality of residuals (assessed using the Shapiro–Wilk test and Q–Q plots), homoscedasticity (assessed by Levene’s test), and multicollinearity (assessed using variance inflation factor) were evaluated. In the linear regression models, the following variables were included as independent predictors of change in CFS, selected based on clinical relevance: age, sex, CPS, hospital LOS, ICU LOS, need for RRT, readmission to hospital, use of rehabilitation and staying in institution at 12 months after inclusion. The regression was adjusted for baseline CFS score. We standardized the covariates to compare effect sizes.

In the logistic regression analysis, we included the following independent predictors of mortality: age, sex, CPS, baseline CFS, Sequential Organ Failure Assessment (SOFA) score on the first day after inclusion, ICU LOS. Risk ratios were estimated using modified Poisson regression with a log-link function and robust standard errors. To facilitate a direct comparison of effect magnitudes across variables with disparate measurement units, continuous predictors were presented as the risk ratio per one standard deviation increase. Overall survival at 12 months was estimated using the Kaplan–Meier method and compared between groups using the log-rank test.

In the overall analysis P-values < 0.05 were deemed statistically significant.

Missing data were not replaced. Loss of data during the follow up was only due to retraction of written consent. If the patient or their proxy did not request deletion of previously collected data, baseline data were retained for analysis.

For statistical analysis we used the R software v.4.3.1 [22].

## Results

**Fig. 1:**
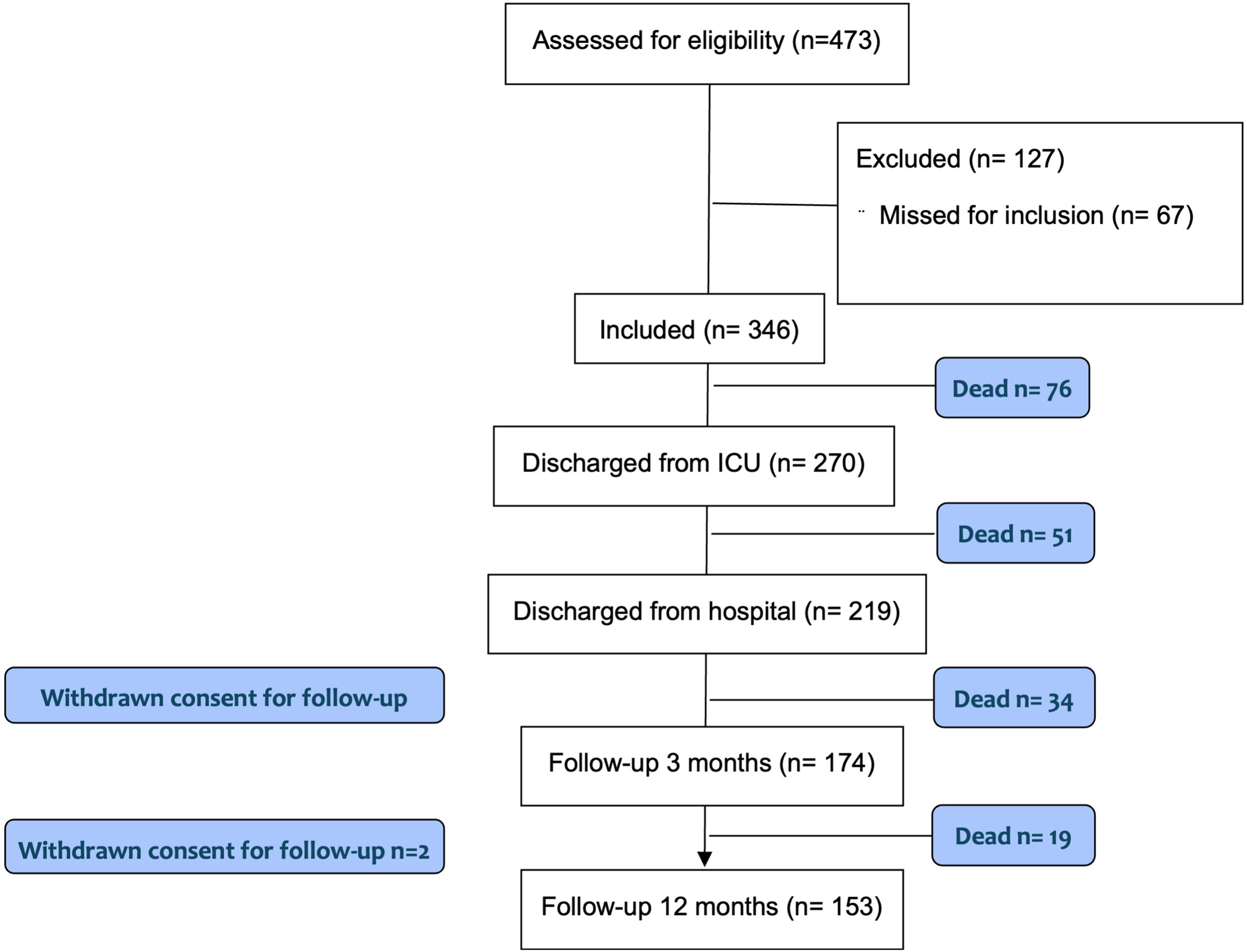
Study Flowchart.

The study enrolled 346 patients after exclusion of 127 individuals, primarily due to missed inclusion (Fig. 1). 153 patients completed follow-up at 12 months.

Baseline and ICU admission characteristics are presented in Table 1. The mean age of the study population was 74 ± 6 years for all CFS-groups. Median CPS was 8 (IQR 7), corresponding to moderate comorbidity. 339 (98 %) lived at home. Of these, 306 patients managed to live at home without help. Treatment limitation was present for 14 patients (4 %) prior to ICU-admission. The most frequent reasons for admission to ICU were sepsis and respiratory failure and 21 % were admitted because of disease or trauma directly affecting the CNS, e.g. ischemic stroke, cerebral haemorrhage and epileptic seizures.

**Table 1:** Baseline, ICU Admission Characteristics and ICU diagnoses.

Variable		n = 346
Demographics	Women, n (%)	117 (34)
	Age, mean (SD)	74 (6)
	BMI, mean (SD)	27 (5)
	Smoking, n (%)	80 (22)
Pre-ICU status	Baseline CFS, median (IQR)	3 (2)
	CPS, median (IQR)	8 (7)
	Residence, home, n (%)	339 (98)
	Treatment limitation, n (%)	14 (4)
Reason for admission	Sepsis, n (%)	68 (20)
	Respiratory failure, n (%)	66 (19)
	Acute surgery, n (%)	55 (16)
	Circulatory failure, n (%)	42 (12)
	CNS disease, n (%)	71 (21)
ICU Diagnoses	Respiratory disease, n (%)	92 (27)
	Circulatory disease, n (%)	77 (22)
	Sepsis, n (%)	45 (13)

The % proportion is calculated from total number of included patients (n = 346).

Abbreviations: SD: standard deviation, IQR: interquartile range, BMI: body mass index, ICU: intensive care unit, CFS: Clinical Frailty Scale, CPS: Comorbidity Polypharmacy Score, CNS: central nervous system.

### Severity of critical illness

Median SOFA score the first day after inclusion was 9 (IQR 4) and was reduced to 2 (IQR 1) from day one to four. 65 patients (19 %) required RRT, 34 (10 %) ICP monitoring, 16 (5 %) prone positioning and 14 (4 %) ECMO. Median ventilator time was 138 (IQR 188 h). Median ICU LOS was 8 days (IQR 9) and median hospital LOS was 19 days (IQR 21). Median CPS was 8 (IQR 7) at baseline, 9 (IQR 7) at three months and 9 (IQR 6) at 12 months. Details for LOS, ventilator time and CPS for different CFS-groups are presented in Table S2 in Supplementary materials.

### CFS trajectory and mortality

**Fig. 2:**
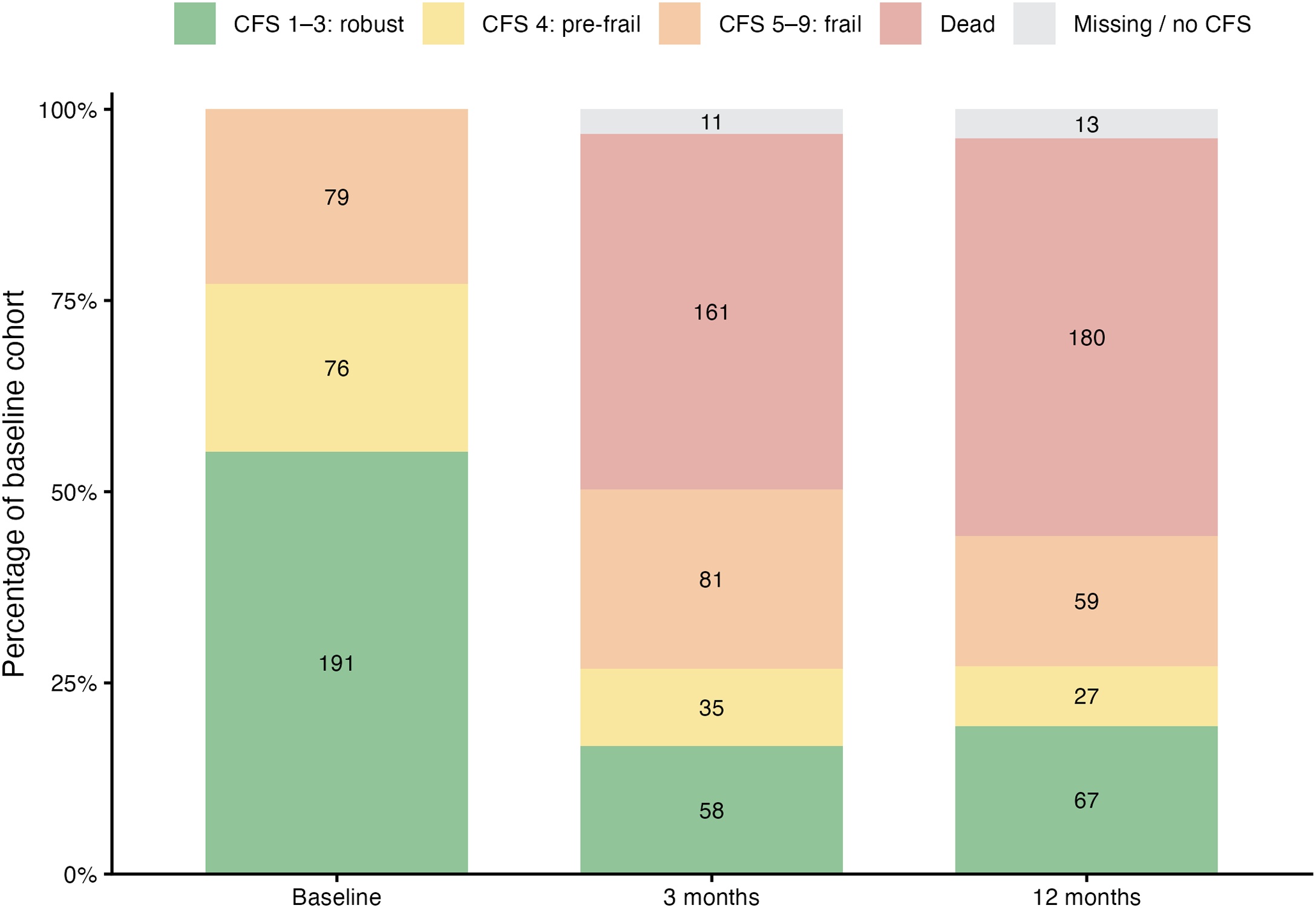
Distribution of CFS-groups among participants at each timeline. Change in frailty status over time according to the Clinical Frailty Scale (CFS). Stacked bars represent the distribution of CFS categories, dead and missing at baseline, 3 months, and 12 months for all included patients (n = 346) at each timepoint. The number of patients contributing to each category is shown in the bars.

CFS score increased significantly from baseline to three and 12 months (*p <* 0.001, paired t test). Prior to critical illness, 191 patients (55 %) were classified robust (CFS 1–3), 76 (22 %) were prefrail (CFS 4) and 79 (23 %) were frail (CFS 5–9) (Fig. 2).

In survivors at three months, the proportion of patients living with frailty was 47%, and at 12 months it was 39 % (Fig. 2). 71 patients (21% of all included patients) were admitted to ICU primarily because of disease or trauma related to the central nervous system (CNS). These patients were at baseline more robust (CFS 1-3, 51/71, 72 %) but were more frail at three (82 %) and 12 months (70 %).

**Fig. 3:**
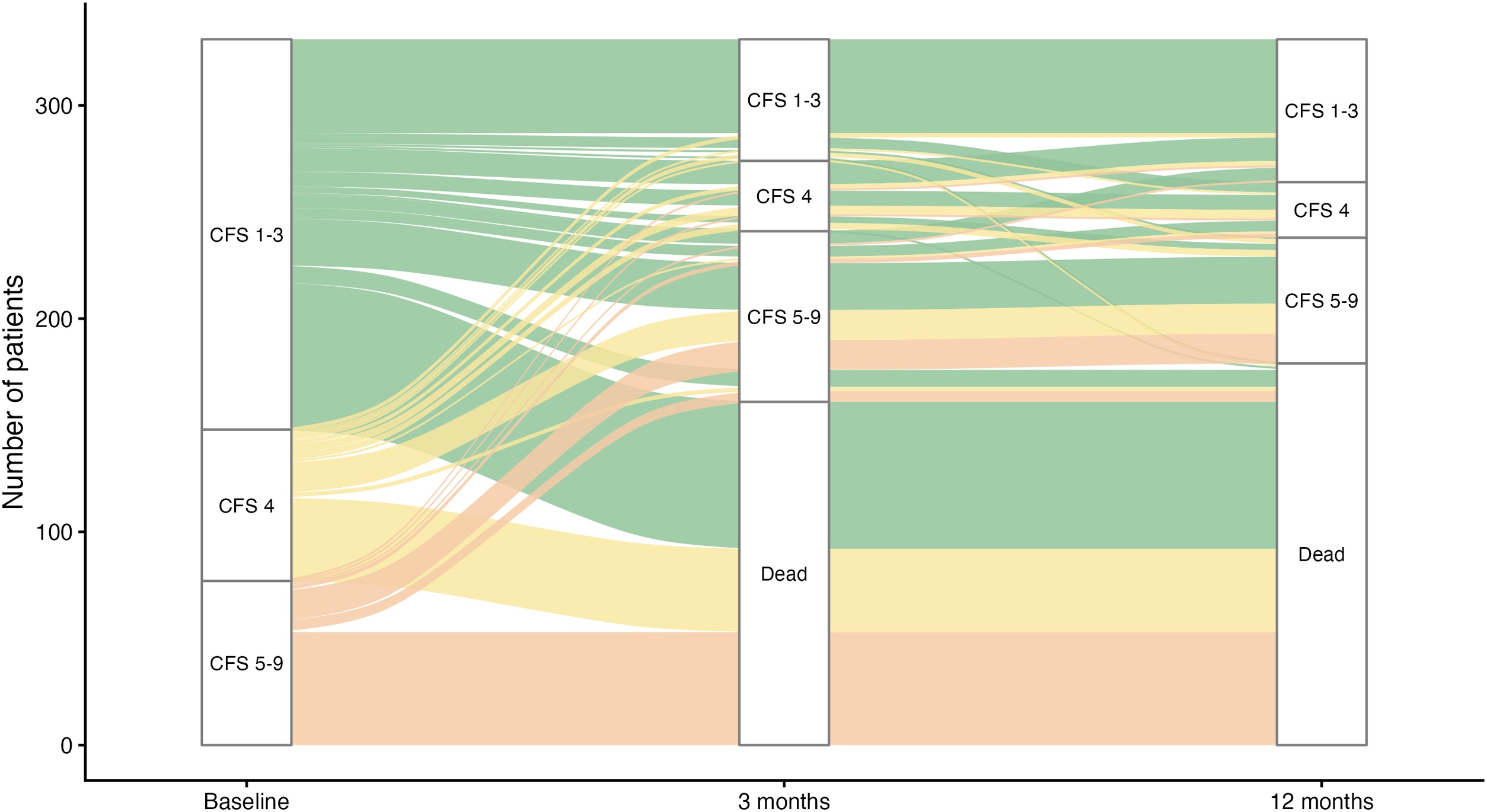
Patient flow between CFS groups. Alluvial plot illustrating change in frailty status over time across Clinical Frailty Scale (CFS) groups including all patients (n = 346). Patients who died before follow-up, flow into the non-survivor group. 153 patients survived to 12 months follow-up.

Detailed CFS-score at each timepoint is presented in Table S1 in supplementary material. Fig. 3 and Table S3 in the supplementary material illustrates individual CFS trajectories. At 12 months, 9 % of the robust patients at baseline deteriorated to prefrail, and 14 % to frail. Of patients being prefrail at baseline, 5 % improved to robust and 25 % ended up frail. Among frail patients at baseline, 3 % improved to robust state, 4 % to prefrail and 18 % remained frail. Among those who survived to 12 months, 71% maintained a stable CFS between three and 12-months, while 19 % showed improvement and 10 % experienced deterioration. Of the 19% who showed improvement, 72 % reached a robust state. Linear regression analysis adjusted for baseline CFS score, showed that the increase in CFS score from baseline to 12 months follow-up was positively associated with rehabilitation (regression coefficient (β 0,3), institutionalisation (β 1,5), ICU-LOS (β 0,3) and negatively with the need for RRT (β −0,4).

The one-year mortality rate was 180/346 (52 %) (Fig. 2). 76 patients (22 %) died in the ICU, and 127 patients (37 %) died before hospital discharge. 161 patients (47 %) died within three months, constituting 89% of all non-survivors (n = 180). The patients with CNS disease had equal mortality as the rest of the cohort.

**Fig. 4:**
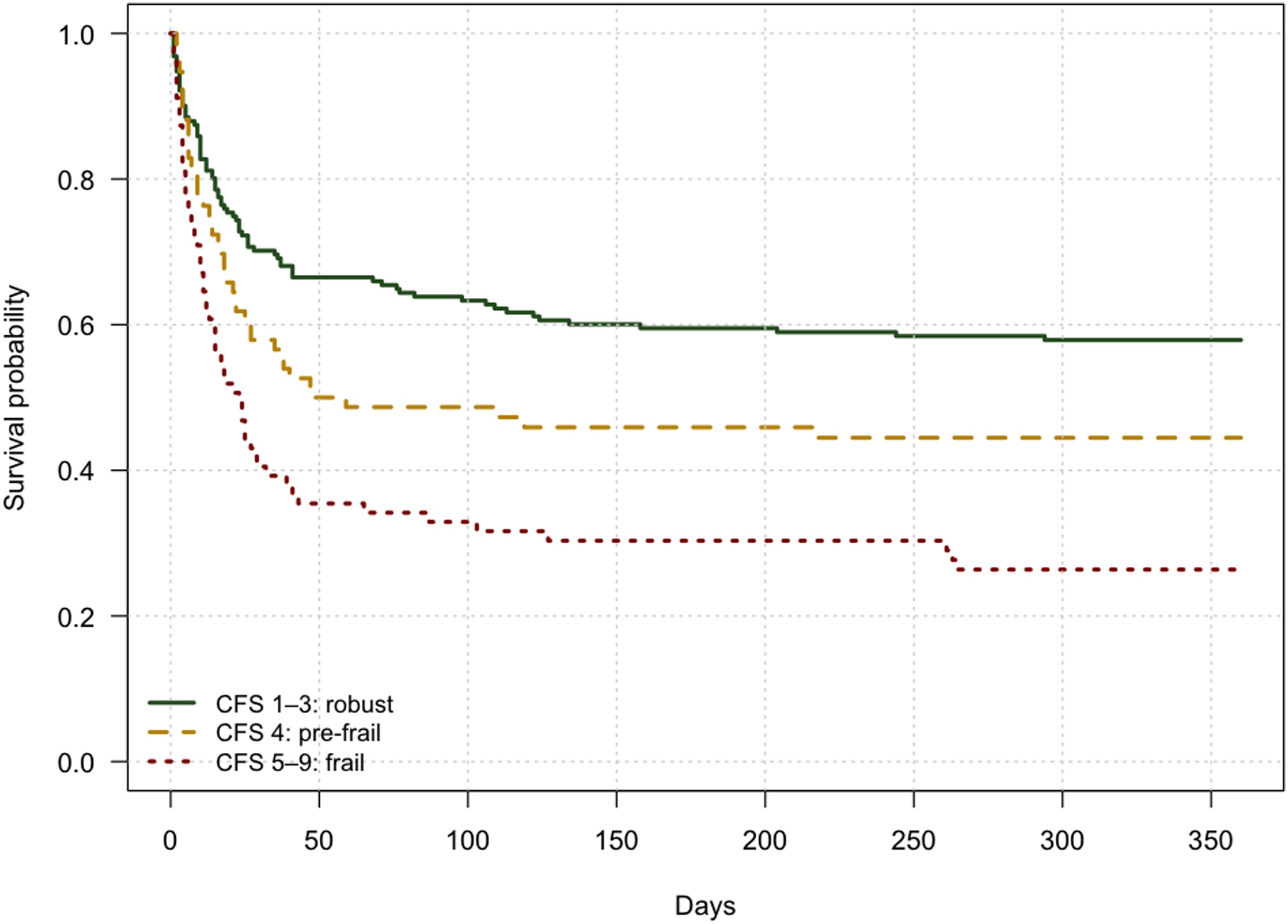
Survival by CFS category. Kaplan–Meier survival curves illustrating survival probability among patients stratified by baseline CFS-groups. Between-group differences were evaluated using the log-rank test and were highly significant (*p* <  0.001).

**Fig. 5:**
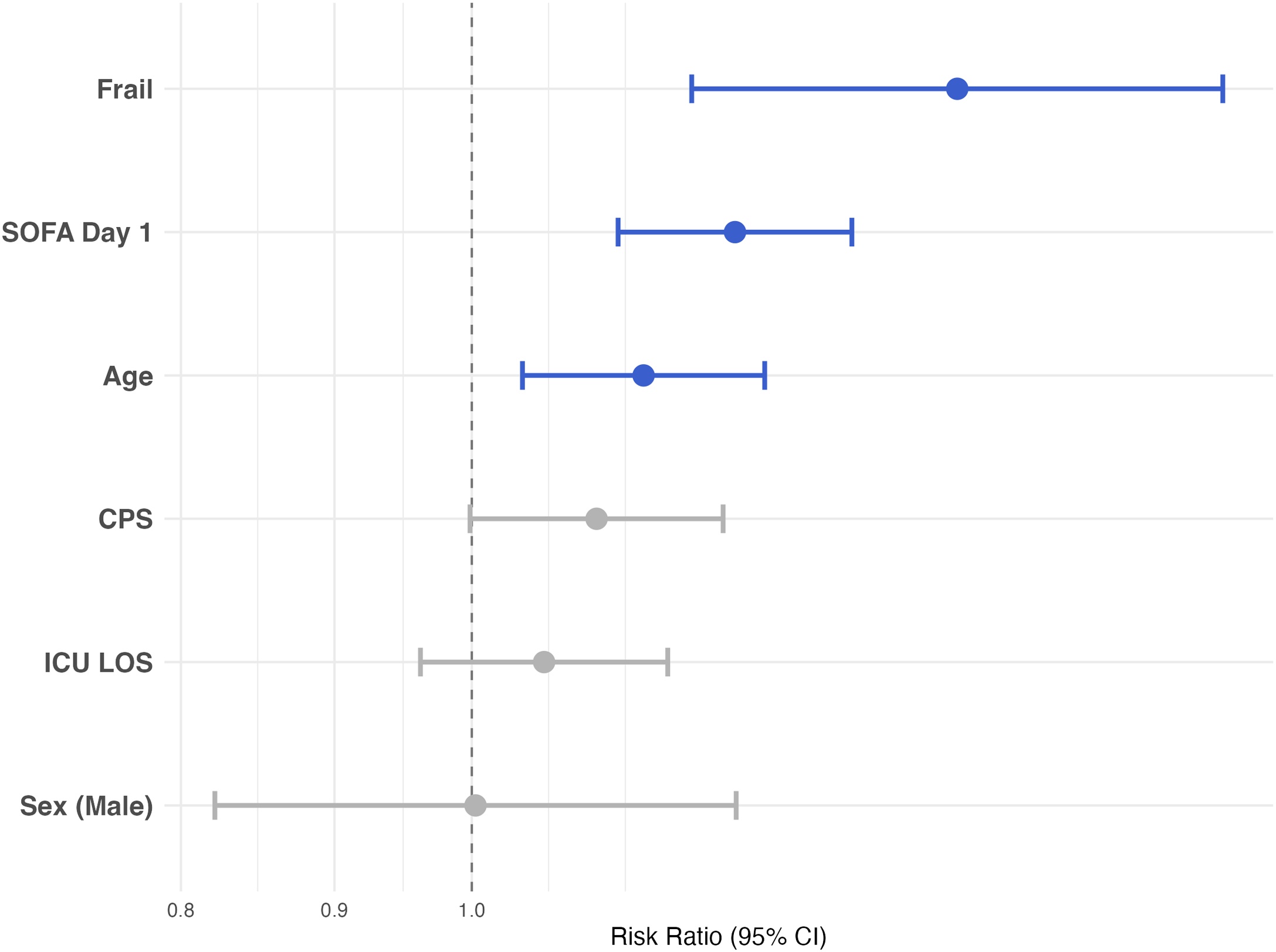
Risk ratio for mortality. Associations between patient characteristics and mortality, presented as Risk Ratio with 95 % Confidence Intervals (CI). Continuous predictors are presented as the Risk Ratio per one standard deviation (SD) increase. Frail is defined as CFS 5–9. The specific SD values employed for scaling in this cohort were as follows: Age (5.6 years), SOFA score (3 points), CPS (5 points), ICU LOS (8.6 days). Abbreviations: CPS, Comorbidity Polypharmacy Score; LOS, length of stay; ICU, intensive care unit; SOFA, Sequential Organ Failure Assessment score.

Kaplan-Meier survival analysis demonstrated that probability of survival was significantly lower among frail patients compared to non-frail (*p <* 0,001) (Fig. 4). Indeed, one-year mortality was 73 % for patients living with frailty at baseline, 55 % for prefrail and 42 % for robust patients (Table S3 in supplementary material). As illustrated in Fig. 5, the strongest predictor for mortality was baseline frailty with 46 % higher risk of death compared to non-frail patients.

### Post-discharge healthcare utilization

By 12 months, 86 % (132/153) of the ICU survivors had returned home. Proportion of patients living at home without care declined from 88 % at admission to 40 % (70/174) at three months but increased to 54 % (83/153) at 12 months. 32 % (49/153) required home care compared to 10 % pre-admission. Of the 219 patients alive at hospital discharge, 79 % were transferred to an institution. At three months, 33 % (58/174) of patients still alive remained in institution, but reduced to 14 % (21/153) at 12 months. 40 % (87/219) of hospital survivors were admitted to a rehabilitation facility during the first year after ICU-treatment. Of these 12 % were admitted at hospital discharge. Among patients receiving rehabilitation, 68 % were robust at baseline, 18 % were prefrail and 14 % were frail. 52 patients had been readmitted to the hospital during the 12-month follow-up. Being frail at 12-month follow-up was significantly associated with living in institution (*p* < 0.01), and rehabilitation stay during the 12 month follow-up period(*p* < 0.001) but not readmissions (*p* = 0.09).

In the exploratory pilot sub-analysis of the final 68 survivors at 12-month follow-up, patients or their relatives were asked to assess the well-being of the ICU survivor compared to the condition prior to the critical illness. 34 respondents reported a decline in their quality of life, while nine indicated an improvement. 13 respondents (19 %) would not want to undergo ICU-treatment again if necessary. Of the 14 respondents who were frail at 12 months follow-up, 10 respondents (71 %) would not want to undergo ICU-treatment again if necessary. Detailed distribution of the CFS scores at baseline and 12-month follow-up for the respondents are presented in Supplemental Table S4.

## Discussion

Despite 98 % of the cohort being community-dwelling prior to ICU admission, our study showed that most elderly ICU patients become more frail or die over a 12-month follow-up period. Pre-critical illness CFS was strongly associated with higher mortality. However, the number of robust patients improved during the period between three and 12 months post discharge. Still, almost one fifth of the patients in the final follow-up reported that they would not want to undergo intensive care treatment again.

### CFS trajectory

As frailty is recognized as an important risk factor for adverse outcomes, and given the ongoing debate regarding ICU triage priorities, it has been suggested that frailty should be considered in all ICU referrals for older patients [4,23] In our study one fourth of the patients included were frail pre-critical illness. This suggests that frailty was not used as an exclusion criterion for ICU treatment in this study. Other studies report a frailty prevalence of 30–45 % among older patients admitted to the ICU, confirming that frailty at present is not a major element in admission decision-making [18,24].

We found that frailty increases from pre-critical illness to three and 12 months follow up. This is also related to the ICU diagnosis as subgroup analysis of CNS diseases demonstrated. On a group level only a minority of mechanically ventilated patients survive and regain the same level of frailty after an ICU stay and is important information for patients and relatives in discussions of whether ICU treatment should be initiated. Our study confirms pre-critical illness frailty to be correlated with worse outcome [7,18].

Interestingly, the prevalence of frailty improved between the three- and 12-month follow-up. This improvement primarily occurred among individuals who were robust prior to ICU admission, experienced a functional decline at three months, and subsequently recovered. Similarly, the number of institutionalized patients decreased over time; nearly all survivors returned home, although requiring more home assistance than they did at baseline. These findings suggest a degree of recovery following the subacute phase of critical illness and indicate that older survivors may require an extended follow-up period to document regained functional capacity. However, these trajectories reflect only the surviving sub-cohort and must be interpreted alongside the high mortality rate observed within the first 3 months. Consistent with our findings, two other studies report at a group level worse frailty one year after ICU admission [25,26]. However, to our knowledge no studies have demonstrated that older patients may be divided in several post ICU trajectories such as death within one-year, extended worse function or only a transitory decline in function.

The use of RRT in our patients was associated with less deterioration of CFS scores in the long term. This observation should be interpreted with caution. Our findings may be subject to confounding by indication, as clinicians will be more prone to initiate organ support such as RRT in patients with a condition of better over-all prognosis. Neither length of mechanical ventilation nor the use of other organ supporting devices was correlated with CFS trajectory and aligns with other critical care studies on frailty that do not report effect of organ support on trajectories [[27], [28]].

In our study, prolonged ICU stay was associated with an increase in CFS scores. While this likely reflects greater baseline illness severity, extended intensive care also exposes vulnerable older adults to severe deconditioning, neurocognitive impairment, nutritional deficits and the risks of a polypharmacy burden. Together, these factors accelerate post-ICU frailty deterioration. Given this high risk of physical and cognitive decline, clinicians must carefully weigh the benefits of prolonged intervention against the potential for harm.

Previous studies indicate that most older ICU survivors express a willingness to undergo ICU treatment again, particularly if survival preserves autonomy and independence [29,30]. The proportion of elderly ICU survivors who would decline future ICU treatment agrees with the result of 19 % in our study. These preliminary results suggest that for some, the post-recovery quality of life does not sufficiently offset the strain of treatment, associated complications and potential long-term sequelae.

### Mortality

Half of the included patients died during the one-year study period. Most deaths occurred before hospital-discharge or in the subacute phase. While data on one-year mortality remain limited, several studies investigating elderly ICU populations have reported a three or six-month mortality rate exceeding 40 % [[31], [32], [33]], though some studies show lower mortality [26,[34], [35], [36]]. This is most likely based on differences in case-mix, since many studies also included non-ventilated patients.

The crucial point is to detect patients facing a poor prognosis at an individual level in advance. Indeed, in our study, baseline CFS scores was the strongest predictor for mortality. The other important factor was SOFA scores the first day after intubation. Both these findings correspond to prior studies on mortality [23,32,33]. Frailty at baseline indicates the resilience and potential of the patient, and SOFA-score the first day after debut of severe critical illness indicates the detrimental effect of the acute illness. It is tempting to pay attention to the combination of these two variables for decision-making to withhold or withdraw ICU treatment. Age is a well-recognized risk factor for ICU mortality [5,34]. However, frailty reflects age-associated deterioration of function across multiple organ systems [37]. Although comorbidity was higher in frail patients at baseline and slightly increased in the follow up, it was not significantly associated with long-term mortality or CFS trajectory in line with some other studies [34,35]. It may be considered that frailty encompass loss of function from age and chronic diseases and as such is a composite measure of the total decline in function. This may explain why frailty might be a better predictive factor compared with age or comorbidity for both short term and long-term mortality among elderly ICU patients [9,14].

### Strengths and limitations

We acknowledge several strengths and limitations of this study. First, by including patients from major centers across all four Norwegian health regions, the cohort provides a national cross section that aligns with major admission trends documented in national registries. Another strength is the near-complete prospective follow-up by few persons. Furthermore, the inclusion of only those requiring mechanical ventilation ensured a strictly critically ill population, distinct from patients admitted solely for close monitoring or with minor organ failures.

While the 12-month follow-up provides valuable insight into long-term outcomes, a limitation of our long-term data is the potential for survival bias. Between three and 12 months, surviving patients largely maintained or improved their frailty status. However, because nearly half of the cohort died within three months, this apparent stabilisation is likely in part a survivorship effect resulting from the high mortality of the frailest patients.

Regarding CFS categorization, while some authors recommend utilizing the full nine-point CFS for evaluation [38], analysing all nine categories requires a substantial sample size to achieve statistical significance, particularly as there are few patients in the most frail groups in critical care. Consequently, we categorized CFS scores into non-frail, pre-frail and frail consistent with the approach adopted in previous large-scale studies [7,18]. These three broad categories are highly practical in fast-paced ICU environments to facilitate rapid risk-stratification, while avoiding unnecessary emphasis on differences within the non-frail spectrum and encompassing an intermediate prefrail category for cases of greater diagnostic uncertainty.

A further limitation is that the CFS was mainly proxy-reported. While proxy reporting may differ from self-reported scores, the CFS has been validated for this purpose [8]. Furthermore, although pre-critical illness CFS was assessed as close to admission as possible, retrospective reporting is inherently susceptible to recall bias. Additionally, while follow-up assessments are prone to observer bias, we attenuated this risk by employing a limited number of trained examiners to conduct both inclusion and follow-up evaluations. We did not assess the inter-rater reliability of the CFS, which has been well-validated in prior studies of elderly critically ill population [8,38].

Finally, we utilized the CPS rather than more validated comorbidity measures like Charlson Comorbidity Index or Elixhauser Index. Consequently, alternative comorbidity indices might yield different results. While the CPS assigns equal weight to all conditions and relies on medication counts as a surrogate—potentially introducing bias—it offers a composite measure of both comorbidity and polypharmacy burden across two dimensions. Furthermore, during telephone interviews, prescription lists are often more easily recalled by patients or proxies than precise diagnostic histories. Additionally, CPS has been used in recent large-scale ICU studies involving elderly patients which facilitate comparison across cohorts [7,33]

### Priorities for Future Research and Knowledge Development

Critical illness in this cohort is marked by high mortality and increased frailty. Future outcome studies in elderly ICU patients should routinely incorporate frailty assessments and extend follow-up beyond three months. Research should identify specific predictors for distinct post-ICU clinical trajectories, such as early mortality, persistent impairment, or subacute recovery. Our data support the utility of reevaluating treatment goals for older ICU patients who fail to improve within the first few days of admission, a strategy often described as a time-limited ICU-trial [38]. Finally, the prevalence of and reasons for dissatisfaction with ICU care, warrant more comprehensive investigation in future research.

## Conclusions

In this Norwegian cohort of patients 65 years or older who have received mechanical ventilation in the ICU, pre-frailty and frailty are common at admission and increase after intensive care treatment. However, patients who survive the subacute phase of three months often improve in terms of functional level and return home, albeit with increased home assistance. Our findings support that baseline CFS assessment and SOFA score the first day after debut of severe critical illness are important predictive variables of long-term functional outcome. We recommend multicentre prospective studies incorporating frailty assessment, extended follow-up, and trajectory-based outcomes to develop individual-level prognostic tools that support shared decision-making around ICU admission.

## Authors' contributions

SKF, BAK and HF conceived and designed the study protocol. SKF and BAK administered the study protocol. RK, PK, DB, OKF, KS contributed to the protocol. SKF, BAK, HF wrote the data analysis plan. BAK, BS, DB, KS, MH, OKF, RK collected the data. BAK and SKF wrote the first draft of the manuscript. All authors contributed to manuscript editing and approved the final version of the manuscript.

## Consent for publication

“Not applicable”.

## Ethics approval and consent to participate

The study was approved by the Norwegian Regional Committee for Medical and Health Research Ethics, (172,784/REK North). Written informed consent was obtained from the patient in the acute phase or at follow up. If the patient was unable to consent, this was retrieved from the proxy. If a patient died in the acute phase before consenting, inclusion was done by deferred consent, according to local regulations.

## Funding

This study was funded by the Northern Norway Regional Health Authority (181021). The funding parties have no influence on the trial design or conduction of the trial.

## Availability of data and material

The datasets used and/or analysed during the current study are available from the corresponding author on reasonable request.

## Declaration of competing interest

The authors declare that they have no competing interests.
