# Supplementary material for: Long-term frailty trajectory in older ICU survivors requiring mechanical ventilation: a prospective cohort study in Norway
Source: Ann Intensive Care. 2026 Jul 16;16:100115. doi: 10.1016/j.aicoj.2026.100115 (PMC13446336; doi:10.1016/j.aicoj.2026.100115)
Supplement: Supplementary file 1 [file mmc1.docx]

Supplementary material

**Long-term trajectory of frailty in elderly ICU survivors**

Kroken BA ^1,2^, Bergum D^3^, Berg KS^4,5^, Hoff M^6^, Fossum OK^6^, Klepstad P^3^, Sjøbø BÅ^7^, Kvåle R^8,9^, Flaatten H^9,10^, Frisvold SK^1,2^

**Affiliation**

^1^ Department of Clinical Medicine, UiT the Arctic University of Norway, Tromso, Norway

^2^ Department of Anaesthesia and Intensive care, University Hospital of North Norway, Tromso, Norway

^3^ Department of Anaesthesiology and Intensive Care Medicine, St Olavs University Hospital

^4^ Department of Anaesthesia and Intensive Care Medicine, Surgical Clinic, Nordland Hospital Trust, Bodø, Norway

^5^ Faculty of Nursing and Health Sciences, Nord University, Bodø, Norway

^6^ Department of Anaesthesia and Intensive care, Akershus University Hospital, Oslo, Norway

^7^ Department of Surgical Services, Intensive Care Unit, Haukeland University Hospital, Bergen, Norway

^8^ Department of Clinical Medicine, University of Bergen, Norway

^9^ Department of Anaesthesia and Intensive care, Haukeland University Hospital; Bergen, Norway

^10^ Department of Research and Development, Haukeland University Hospital, Bergen Norway

**Corresponding author**

Shirin Kordasti Frisvold, shirin.frisvold@uit.no

**Table S1. CFS distribution through study-phases**

| Variable | Baseline  n=346 | 3 months  n=174 | 12 months  n=153 |
| --- | --- | --- | --- |
| CFS 1, n (%) | 36 (10) | 6 (3) | 8 (5) |
| CFS 2, n (%) | 58 (17) | 19 (11) | 21 (14) |
| CFS 3, n (%) | 97 (28) | 33 (19)’ | 38 (25) |
| CFS 4, n (%) | 76 (22) | 35 (20) | 27 (18) |
| CFS 5, n (%) | 37 (11) | 25 (14) | 16 (10) |
| CFS 6, n (%) | 33 (10) | 19 (11) | 17 (11) |
| CFS 7, n (%) | 7 (2) | 29 (17) | 25 (16) |
| CFS 8, n (%) | 1 (0) | 7 (4) | 1 (1) |
| CFS 9, n (%) | 1 (0) | 1 (1) | - |
| CFS median (IQR) | 3 (2-4) | 4 (3-6) | 4 (3-6) |

Percentage proportion is calculated from survivors at actual timepoint.

Abbreviations: CFS: Clinical Frailty Scale, IQR: interquartile range

centage

**Table S2. Length of stay in ICU and hospital, time on ventilator and CPS, based on CFS-groups at baseline**

| Baseline CFS-group | CFS 1-3  n = 191 | CFS 4  n = 76 | CFS 5-9  n = 79 |
| --- | --- | --- | --- |
| LOS ICU (days), median (IQR) | 9 (9) | 8 (9) | 7 (9) |
| LOS hospital (days), median (IQR) | 19 (23) | 19 (17) | 17 (19) |
| Time Ventilator (hours), Median (IQR) | 153 (228) | 129 (145) | 124 (146) |
| CPS median (IQR) | 7 (6) | 9 (6) | 12 (7) |
|  |  |  |  |
| Abbreviations: CFS: Clinical Frailty Scale, LOS: Length of stay, ICU: intensive care unit, CPS: Comorbidity Polypharmacy Score, IQR: interquartile range | | | |

**Table S3a. CFS-trajectory based on CFS-groups at baseline**

| **CFS-group**  **baseline** | **CFS-group**  **12 months** | **Count**  **n (%)** |
| --- | --- | --- |
| CFS 1-3 | Dead | 80 (42) |
| CFS 1-3 | CFS 1-3 | 61 (32) |
| CFS 1-3 | CFS 4 | 17 (9) |
| CFS 1-3 | CFS 5-9 | 26 (14) |
| CFS 1-3 | Consent withdrawn | 7 (4) |
| CFS 4 | Dead | 42 (55) |
| CFS 4 | CFS 1-3 | 4 (5) |
| CFS 4 | CFS 4 | 7 (9) |
| CFS 4 | CFS 5-9 | 19 (25) |
| CFS 4 | Consent withdrawn | 4 (5) |
|  |  |  |
| CFS 5-9 | Dead | 58 (73) |
| CFS 5-9 | CFS 1-3 | 2 (3) |
| CFS 5-9 | CFS 4 | 3 (4) |
| CFS 5-9 | CFS 5-9 | 14 (18) |
| CFS 5-9 | Consent withdrawn | 2 (3) |
|  |  |  |

Percentage proportion is calculated from number of patients in actual CFS-group at baseline.

Abbreviations: CFS: Clinical Frailty Scale

**Table S3b. CFS-trajectory based on CFS-groups for survivors at 3 month follow-up**

| **CFS-group**  **3 months** | **CFS-group**  **12 months** | **Count**  **n (%)** |
| --- | --- | --- |
|  |  |  |
| CFS 1-3 | Dead | 2 (3) |
| CFS 1-3 | CFS 1-3 | 46 (79) |
| CFS 1-3 | CFS 4 | 6 (11) |
| CFS 1-3 | CFS 5-9 | 3 (5) |
| CFS 1-3 | Consent withdrawn | 1 (2) |
| CFS 4 | Dead | 1 (3) |
| CFS 4 | CFS 1-3 | 14 (40) |
| CFS 4 | CFS 4 | 12 (34) |
| CFS 4 | CFS 5-9 | 6 (17) |
| CFS 4 | Consent withdrawn | 2 (6) |
| CFS 5-9 | Dead | 15 (18) |
| CFS 5-9 | CFS 1-3 | 7 (9) |
| CFS 5-9 | CFS 4 | 8 (10) |
| CFS 5-9 | CFS 5-9 | 50 (62) |
| CFS 5-9 | Consent withdrawn | 1 (1) |
|  |  |  |

Percentage proportion is calculated from number of patients in actual CFS-group at 3 months

follow-up. Abbreviations: CFS: Clinical Frailty Scale

**Table S4. Preference if future need for new ICU-treatment should arise**

| Preference if repeat | Baseline  CFS-group | Outcome  CFS-group | Count  n (%) | Total in group  n |
| --- | --- | --- | --- | --- |
|  |  |  |  |  |
| Refuse ICU-treatment, n (%) | CFS 1-3 | CFS 1-3 | 3 (23) | 13 |
| Refuse ICU-treatment, n (%) | CFS 1-3 | CFS 5-9 | 4 (31) | 13 |
| Refuse ICU-treatment, n (%) | CFS 4 | CFS 5-9 | 3 (23) | 13 |
| Refuse ICU-treatment, n (%) | CFS 5-9 | CFS 5-9 | 3 (23) | 13 |
|  |  |  |  |  |
| Want ICU-treatment, n (%) | CFS 1-3 | CFS 1-3 | 23 (42) | 55 |
| Want ICU-treatment, n (%) | CFS 1-3 | CFS 4 | 7 (13) | 55 |
| Want ICU-treatment, n (%) | CFS 1-3 | CFS 5-9 | 9 (16) | 55 |
| Want ICU-treatment, n (%) | CFS 4 | CFS 1-3 | 2 (4) | 55 |
| Want ICU-treatment, n (%) | CFS 4 | CFS 4 | 6 (11) | 55 |
| Want ICU-treatment, n (%) | CFS 4 | CFS 5-9 | 3 (5) | 55 |
| Want ICU-treatment, n (%) | CFS 5-9 | CFS 4 | 3 (5) | 55 |
| Want ICU-treatment, n (%) | CFS 5-9 | CFS 5-9 | 2 (4) | 55 |

Abbreviations: CFS: Clinical Frailty Scale, ICU: intensive care unit

1
